# Support for Smoke-Free Multi-Unit Housing Policies among Racially and Ethnically Diverse, low-Income Seniors in South Florida

**DOI:** 10.1007/s10823-014-9247-4

**Published:** 2014-10-28

**Authors:** Nicole J. Cook, Lucas Hollar, Summer Chavez, David L. Quinn, Teina Phillips, Michael DeLucca, Lindsay Corrales

**Affiliations:** 1 Master of Public Health Program, College of Osteopathic Medicine, Nova Southeastern University, 3200 South University Drive, Fort Lauderdale, FL 33328 USA; 2 Broward Regional Health Planning Council, Hollywood, FL USA

**Keywords:** Culturally diverse, Low-income, Multi-unit housing, Elders, Smoke-free policy, Tobacco-free

## Abstract

Previous studies have gauged support for implementing smoke-free multi-unit housing (MUH) policies in the United States, but none have specifically examined attitudes among racially and ethnically diverse elders living in low-income MUH. We surveyed a convenience sample of elders 62 years of age and older (*n* = 807) across 24 low-income housing properties in Broward County, Florida in order to assess residents’ smoking behaviors, exposure to second-hand smoke, and support for smoke-free policies. The study sample was ethnically and racially diverse with Hispanics comprising more than 61 % of the population, and 22 % identifying as Black or other races. Although close to 22 % of the sample were former smokers, only 9 % of residents reported being current smokers. The majority of residents surveyed supported no-smoking policies: 75 % support no-smoking policies for individual units; 77 % supported no-smoking policies in common areas; and, 68 % supported no-smoking policies in outdoor areas. Over 29 % of residents surveyed reported being exposed to secondhand smoke entering their units from elsewhere in their building. Residents who reported having a home smoking rule were more than twice as likely to support an indoor policy compared to residents who allowed smoking anywhere in their home (OR = 2.36; 95%CI 1.25–4.43; *p* ≤ 0.01), and nonsmoking residents were nearly three times as likely to support an indoor policy compared to smokers (OR = 2.89; 95%CI 1.44–5.79; *p* ≤ 0.01). Support for an indoor policy was not modified by age, gender, ethnicity or race. . This study demonstrates that elders living in low-income MUH properties overwhelmingly supported the implementation of smoke-free policies.

## Introduction

There is no level of SHS that is recognized to be safe (CDC 2011; USDHHS 2006, 2014). Residents living in multi-unit housing (MUH) with smokers are vulnerable to the detrimental effects of secondhand smoke (SHS). As SHS exposure is higher among people who are low-income and from racial minorities (CDC 2010), creating smoke-free living environments in diverse low-income and subsidized housing communities is an important public health strategy for reducing serious health hazards associated with SHS exposure.

Low-income elders have a unique risk of the sequelae of SHS exposure including exacerbation of bronchitis, pneumonia, cancer, and cardiovascular disease (Helburn 2007). In addition, SHS has been linked to an increased risk of dementia in the elderly, especially those with pre-existing cardiovascular disease (Barnes et al. 2010; Barrett 2007). SHS can also exacerbate chronic obstructive pulmonary disease (COPD) (Eisner et al. 2009) and increase the risk of psychological distress (Hamer et al. 2010). Non-smokers who are exposed to SHS are at increased risk of premature death and increase their risk of developing lung cancer by 20–30% (USDHHS 2014).

Involuntarily, residents of MUH complexes might be exposed to higher levels of SHS due to building factors, such as shared air between units, units of reduced size and low levels of ventilation (Kraev et al. 2009). It has been demonstrated that smoke from apartments where there are smokers can leak into hallways and other apartments in the same building, leading to an increase in nicotine levels among non-smokers (Kraev et al. 2009). SHS can also travel from the outdoors, such as balconies or open windows, into non-smoker apartments through windows and ventilation systems (Helburn 2007; Pizacani et al. 2011). It has been estimated that there may be up to 65 % air exchange between apartment units in the same building (Helburn 2007).

No-smoking policies can reduce SHS exposure, thereby improving health (USDHHS 2014; Heffernan and O’Neill 2013; EPA 1992; Vozoris and Lougheed 2008; Pizacani et al. 2012) and reducing smoke-related property damage (HUD 2012). King et al. projected that prohibiting smoking in subsidized housing would save approximately $521 million per year overall. The breakdown of savings includes $341 million in SHS-related healthcare expenditures, $108 million in renovation expenses, and $72 million in smoking-attributable fire losses (King et al. 2013).

Efforts to implement smoke-free MUH are gaining momentum across the United States both at the state level and at the municipal level. In 2012, the US Department of Housing and Urban Development (HUD) reissued a 2009 notice encouraging Public Housing Authorities (PHA) to implement non-smoking policies in housing units (HUD 2012). As of October 2013, more than 180 municipalities in 32 states had implemented smoke-free MUH laws or policies that restrict smoking in public and affordable MUH units (ANRF 2013).

In many communities, stakeholders of smoke-free MUH surveyed residents in order to understand support and barriers for smoke-free MUH policy development and implementation. Surveys with residents in Oregon, Minnesota, Ohio and California have shown consistently that a majority of residents support smoke-free living (Pizacani et al. 2012; Hewett et al 2007; Hood et al. 2013; Baezconde-Garbanati et al 2011). However, none of these studies focused exclusively on elders living in MUH.

Our study is unique in that we surveyed a large racially and ethnically diverse population of elders living in low-income MUH housing in order to understand current smoking behaviors and exposure to second-hand smoke and to determine support for no-smoking policies.

## Methods

### Setting

Broward County, FL, the 18th largest county in the United States, has nearly 400,000 MUH units. With funding from the Centers for Disease Control and Prevention, Community Transformation Grant (CTG), which granted 61 awards in 36 states from 2011 to 2014, community stakeholders worked towards implementing smoke-free MUH policies, with an initial focus on low-income senior housing (USDHHS 2013). When the project was initiated, there were no public or low-income MUH complexes with smoke-free policies in place in the County.

### Study Population

Public and low-income housing properties and housing properties operating in low-income zip codes were targeted for surveying between March 2013 and September 2013. Utilizing a convenience sample, properties were contacted by community stakeholders, including the American Lung Association, the Florida Department of Health in Broward County, and Nova Southeastern University Master of Public Health Program to identify their willingness to participate in the survey. Of the 24 properties surveyed and included in this study, 18 properties were HUD subsidized low-income senior properties, five were public housing authority managed properties and one was a low-income market-rate property (not senior-specific). e followed HUD age guidelines for low-income senior housing eligibility and included all elders 62 years of age and older in our study.

### Survey Instrument and Measures

The smoke-free MUH survey included 19 questions that captured demographic characteristics of residents and assessed residents’ smoking behaviors, exposure to SHS, and support for smoke-free MUH policies. Our survey was a modified version of the “MUH Resident Survey,” developed by a group of experts for use by the CDC’s Division of Community Health (DCH) CTG awardees (DCH National Evaluation Team 2012). Questions from the DCH CTG survey were adapted from existing interview measures developed by Roswell Park Cancer Institute, CDC, and the New York City Housing Authority. The tool was translated into Spanish and Haitian Creole by native-speaking students from Nova Southeastern University Master of Public Health Program. After initial translation, the survey was reviewed by other native speakers from outside the Master of Public Health program, including property managers, social service coordinators and community stakeholders. The survey was pilot tested prior to administration.

#### Policy-Items

We assessed attitudes towards smoke-free policies (our dependent variable) through three questions: “To what extent do you support a no-smoking policy in YOUR building for all individual apartments?” “To what extent do you support a no-smoking policy in YOUR building for all common areas (such as hallways, lobby, laundry room, stairwells, garage or lounge/party room?)” and “To what extent do you support a no-smoking policy in YOUR building for all outdoor areas (such as courtyards, yards, swimming pools, and children’s play areas?” Responses included, “Support, Do NOT support, Don’t know/not sure.”

#### SHS Exposure Items

We ascertained current exposure to SHS by asking, “How often does tobacco smoke enter your own apartment from somewhere else in or around your building?” Responses items were “Everyday, Sometimes, Never and Don’t know/Not sure.” For analysis we created a new dichotomous variable to compare “Every day” and “sometimes” verses “Never.”

#### Current Smoking Behaviors and Home Smoking Rules

We assessed smoking behaviors by asking, “Do you NOW smoke cigarettes every day, some days or not at all?” To understand residents’ current home smoking rules, we asked residents if they allow smoking anywhere in their home, some places in their home or not at all in their home. For analysis, we combined responses to create dichotomous variables assessing “smoker” verses “non-smoker” and “smoking allowed in home” verses “smoking not allowed in home.”

#### Tenant Demographics and Health co-Morbidities

Tenant demographics were assessed through questions asking the tenant their primary language, gender, age, ethnicity, race, educational attainment, how long they lived in their apartment. We also assessed self-reported comorbidities among the tenant and other residents in the unit by asking “Does anyone living in your apartment have any of the following illnesses: Asthma, Lung Disease (such as chronic bronchitis or COPD), Heart Disease, Cancer.”

### Survey Administration

Residents in 22 of the 24 properties were surveyed as part of resident events; all residents were invited to come at an advertised time to the properties’ social or recreation hall, and the survey was administered in-person by community partners and public health graduate students to all interested tenants. Two properties were surveyed by the property manager leaving flyers on the residents’ doors and asking residents to come complete the survey in the property manager’s office. Across the 24 properties, the overall response rate was 23.1 %, ranging from a high of 100 % to a low of 2 %. The median response rate for properties in this study was 25.7 % across the 24 properties. In 15 of the sites, residents were encouraged to join the events and complete the survey in order to receive a raffle ticket for small prizes ($5 or less.)

Initially, all surveyors were trained by the PI prior to survey administration. As the surveying continued throughout Spring 2013, a trained surveyor and community stakeholder from either the American Lung Association, the Florida Department of Health in Broward County, or Nova Southeastern University Master of Public Health Program conducted the training prior to each survey event. Additionally, a survey procedure guide was distributed to new surveyors to help reduce interview bias.

### Statistical Analysis

Descriptive statistics were used to describe the surveyed population in terms of demographic characteristics and support for smoking policies. Subgroup analyses were performed using Pearson chi-square, two-tailed tests (*p* < 0.05) to assess differences in support for policies by demographic characteristics of interest, current smoking behavior, exposure to SHS, smoking related co-morbidities and home smoking rule.

To assess predictors of support for an indoor no-smoking policy, we conducted multivariate modeling using binary logistic regression to examine if significant variables in the bivariate analysis, including current smoking behavior and home smoking rules, were mediated by demographic characteristics. We tested for multi-collinearity and interaction terms among variables selected for the adjusted model. The final model was adjusted for age group, gender, ethnicity, race, home smoking rule and current smoking status. All analyses were conducted in SPSS Ver. 22, and a *P* value of less than 0.05 was deemed statistically significant.

The research was approved as exempt by Nova Southeastern University’s Institutional Review Board.

## Results

Most respondents in our convenience sample were women (77.0 %.) In terms of age group, 72.6 % of the sample was between 70 and 89 years old. Only 30.5 % of the sample listed English as their primary language, with 57 % listing Spanish as their primary language and 8.1 % listing Creole or French. The sample was ethnically and racially diverse, with 60.8 % self-reporting as Hispanic and 22.2 % of the population self-reporting as Black or other races. In terms of education, 39 % of the residents surveyed had not graduated high school, and only 11.5 % were college graduates (Table 1).

**Table 1:** Characteristics of MUH survey population, ≥62 years of age (*n* = 807)

	No. of residents	Column %
Gender		
Male	168	20.8
Female	621	77.0
Unreported	18	2.2
Age group		
<=69	127	15.7
70–79	334	41.4
80–89	252	31.2
90+	57	4.6
Unreported	37	4.6
Primary Language		
English	246	30.5
Spanish	464	57.5
Creole	65	8.1
Other	32	4.0
Race		
Black	172	21.3
White	569	70.5
Other race	8	0.9
Don’t know/Unreported	58	7.2
Ethnicity		
Hispanic	491	60.8
Non-Hispanic	276	34.2
Don’t know/Unreported	40	4.9
Education		
Less than high school	159	19.7
Some high school	151	18.7
High School grad	223	27.6
Some college/technical	166	20.6
College grad	93	11.5
Don’t know/Unreported	15	1.9

Nearly 22 % of respondents in our sample were former smokers (smoked at least 100 cigarettes in their lifetime), but only 9.3 % were current smokers. More than 29 % (*n* = 236) of residents surveyed said they were exposed to SHS entering their units from somewhere else in or around their building. Among respondents, 15.2 % reported having asthma, 12.3 % lung disease (such as chronic bronchitis or COPD), 18.6 % heart disease, and 8.1 % cancer (all kinds included). Most residents (77.6 %) reported they had a home smoking rule that did not allow smoking anywhere in their homes (Table 2).

**Table 2:** Reported smoking rates, exposure to second-hand smoke and comorbidity of asthma, lung disease, heart disease or cancer among elder residents of low-income housing properties (*n* = 807)

	No. of residents	% of total respondents
Current smoking behaviors		
Current smoker	75	9.3
Former smoker	177	21.9
Exposure to second-hand smoke		
Everyday	79	9.8
Some days	157	19.5
Not at all	523	64.8
Don’t know	42	5.2
Unreported	6	0.7
Smoking related Co morbidity		
Asthma	123	15.2
Lung Disease	99	12.3
Heart Disease	150	18.6
Cancer	65	8.1
Home smoking rule		
Not allowed	626	77.6
Sometimes allowed	35	4.3
Always allowed	62	7.7
Don’t know	69	8.6
Unreported	15	1.9
All Residents	807	

Overall, the majority of residents supported no-smoking policies: 75.2 % supported no-smoking policies for individual units; 76.8 % supported no-smoking policies in common areas (such as hallways, laundry room, lobbies), and 67.9 % supported no-smoking policies in outdoor areas such as courtyards.

There were no significant differences in support for no-smoking policies of any kind by race, exposure to SHS, or presence of a smoking-related comorbidity(ies). Hispanic residents, older residents, females and residents who do not allow smoking in their home were significantly more likely to support both indoor and outdoor smoking policies. (*p* ≤ 0.05.). Current smokers were significantly less likely to support a no-smoking indoor individual apartment policy or a no-smoking outdoor policy, but there was no difference in support of a common area policy for smokers compared to non-smokers (Table 3).

**Table 3:** Comparison of support for smoking policies by resident characteristics among elder residents of low-income housing properties^a^

	No. of residents	% support indoor	P value^b^	% support common	P value^b^	% support outdoor	P value^b^
Gender			0.012		0.086		0.014
Male	157	73.2		77.6		59.6	
Female	568	82.3		83.5		71.1	
Age group			0.039		0.678		0.022
< =69	113	75.2		80.3		57.6	
70–79	315	77.8		81.5		66.9	
80–89	241	84.2		82.8		73.8	
> =90	56	89.3		87.5		80.7	
Primary Language			<0.001		0.011		<0.001
English	222	70.7		75.8		60.1	
Spanish	447	83.4		84.1		79.2	
Creole	62	82.3		81.0		80.6	
Other	27	96.3		96.4		92.0	
Race			0.660		0.460		0.511
Black	156	81.4		79.9		72.1	
White	540	79.8		82.4		74.7	
Ethnicity			0.015		0.314		0.001
Hispanic	4	83.0		83.2		78.2	
Non-Hispanic	253	75.5		80.2		66.5	
Current smoking behavior			<0.001		0.829		<0.001
Smoker	60	51.7		81.0		40.6	
Non smoker	677	82.3		82.0		77.3	
Exposure to SHS			0.471		0.150		0.788
Currently exposed	215	81.9		79.2		73.5	.
Not exposed	498	79.5		83.6		74.4	
Smoking related co-morbidities^c^			0.632		0.676		0.883
One or more co- morbidity	238	81.0		82.5		74.0	
No co-morbidity	369	79.5		81.3		74.4	
Home Smoking rule			0.000		0.741		0.000
Allowed	84	65.5		84.3		59.6	
Not allowed	605	82.6		82.9		77.8	

^a^ “Don’t know” and missing values removed from analysis for each comparison

^b^Pearson chi-square

^c^ Comorbidity includes Asthma, Lung Disease, Heart Disease and/or cancer

We modeled support for an indoor no-smoking policy for all individual apartments in the respondents' buildings using logistic regression. Our final adjusted model included age, gender, ethnicity, current smoker and home smoking rule. Residents who reported having a smoking rule were more than twice as likely to support an indoor policy compared to resident who allow smoking anywhere in their home (OR = 2.36; 95%CI 1.25–4.43; *p* ≤ 0.01.) Nonsmoking residents were nearly three times as likely to support an indoor policy compared to smokers (OR = 2.89; 95%CI 1.44–5.79; *p* ≤ 0.01). Age, gender, ethnicity and race were not significant contributors towards explaining support for an indoor no-smoking policy in the final model (Table 4).

**Table 4:** Predictors of support for indoor smoking policy among low-income elders^a^

Predictor	Support for Indoor Smoking Policy
Age group	
<=69	1.0
70–70	1.24 (0.67–2.29)
81–89	0.843 (0.43–1.65)
> = 90	0.37 (0.10–1.39)
Gender	
Male	1.0
Female	0.1.50 (0.903–2.46)
Ethnicity	
Hispanic	1.0
Non-Hispanic	0.61 (0.369–1.00)
Race	
White	1.0
Black	1.42 (0.78–1.61)
Home smoking rule Allowed	1.0
Not allowed	2.36 (1.25–4.43) ^b^
Current smoking behavior	
Non -smoker	1.0
Smoker	2.89 (1.44–5.79) ^b^

^a^OR = odds ratio; CI = confidence interval. OR estimates are based on the logistic regression model which included age, gender, race, ethnicity, current smoker and home smoking rule. ORs are considered significant if the 95 % CI does not include 1.0

^b^ *p* ≤ 0.01

## Discussion

Although there are other studies that have assessed support for smoke-free MUH in the United States, the work we present here is the first study to examine attitudes toward implementing smoke-free housing policies among a large group of racially and ethnically diverse elders living in low-income MUH. In our study, a clear majority of elders living in the low-income housing properties surveyed support no-smoking policies for individual apartments, common areas and outdoor areas. Overall, our findings are consistent with other studies exploring support for smoke-free MUH policies among residents which ranged from 42 to 79 % support (Ballor et al. 2013; Hennrikus et al. 2003; Baezconde-Garbanati et al. 2011; Hewett et al. 2012; Licht et al. 2012; King 2010). After adjusting for population characteristics, there were no differences among support for prohibitive smoking policies by exposure to SHS, smoking-related comorbidity (is) or race. As expected, smokers had a lower level of support for non-smoking policies than smokers, but a large percentage of smokers did support indoor and common area no-smoking policies.

With regards to our findings by race and ethnicity, we found that there was no difference in support for blacks verses whites or Hispanics verses non-Hispanics. This is relevant and important information in Broward County, which is home to a diverse population of elders from the Caribbean Islands and South America. The large sample size further suggests that low-income elders are in favor of smoke-free MUH policies, and it offers support for ongoing efforts by community partners to work with property owners and managers to continue to implement smoke-free MUH policies. Following surveying and dialogue among community partners, fifteen of the properties surveyed subsequently adopted prohibitive smoke-free MUH policies in October, 2013. Community partners noted that results from this large survey, which have been shared across numerous forums in Broward County, are a useful tool for them as they continue to build partnerships with property managers and owners interested in smoke-free MUH. Applying these results in ongoing work demonstrates knowledge of the local context, an important strategic step for partners working actively on developing and implementing smoke-free MUH (Satturlund et al. 2013).

The overarching goal of implementing smoke-free policies is to create healthy living environments by reducing smoking in areas where non-smokers can be exposed to SHS. Therefore, communities working towards smoke free MUH must be cognizant of meeting both smoker and non-smoker needs throughout the policy development and implementation process. While implementation of smoke-free policies have shown associated increases in cessation-related behaviors (Pizacani et al. 2012; USDHHS 2014), the implementation of policies should not be punitive towards smokers. In Broward County, Florida smoking cessation partners were involved throughout the policy development process, and smoking cessation groups were provided, and continue to be provided, at a number of properties included in this study. To ensure that the policy did not alienate residents, regardless of smoking behaviors, a smoking designated area, at least 25 f. away from entrances, windows and ventilations systems, was constructed at each property that implemented a smoke-free policy.

Limitations of this study include possible selection bias, due to the convenience sampling approach as well as low response rates at some of the properties surveyed. Other limitations include possible information bias, due to the nature of self-report surveys and the potential for socially desirable responses. In addition, as a gateway city to South America and the Caribbean Islands, Broward County, Florida is diverse; over 28 % of the population is Black or African American, and 27 % is Latino/Hispanic. More than 31 % of residents were foreign born (United States Census Bureau 2013). Given that our study included a diverse, low-income population, results may not be generalizable to less diverse communities or higher income communities.

## Conclusion

This study is the first to evaluate levels of support for smoke-free policies among a large population of low-income racially and ethnically diverse elders living in low-income MUH properties. Findings demonstrate that elders living in low-income MUH properties overwhelmingly support smoke-free policies. As elders living in MUH can have serious health consequences due to SHS exposure, public health practitioners, property managers and residents must build on current momentum and continue to work together to foster healthy living environments for our elders by developing and implementing smoke-free policies.
